# RETRACTION: Effect of Body Mass Index on Surgical Site Wound Infection, Mortality, and Postoperative Hospital Stay in Subjects Undergoing Possibly Curative Surgery for Colorectal Cancer: A Meta‐Analysis

**DOI:** 10.1111/iwj.70378

**Published:** 2025-03-21

## Abstract

**RETRACTION:** QiaoY. , ZhangT. , BaiT. , PengX. , LinH. , and ZhangA. , “Effect of Body Mass Index on Surgical Site Wound Infection, Mortality, and Postoperative Hospital Stay in Subjects Undergoing Possibly Curative Surgery for Colorectal Cancer: A Meta‐Analysis,” International Wound Journal 20, no. 1 (2023): 164–172, 10.1111/iwj.13860.35670494 PMC9797934

The above article, published online on 07 June 2022, in Wiley Online Library (http://onlinelibrary.wiley.com/), has been retracted by agreement between the journal Editor in Chief, Professor Keith Harding; and John Wiley & Sons Ltd. Following an investigation by the publisher, all parties have concluded that this article was accepted solely on the basis of a compromised peer review process. The editors have therefore decided to retract the article. The authors did not respond to our notice regarding the retraction.

**RETRACTION:** Y. Qiao , T. Zhang , T. Bai , X. Peng , H. Lin , and A. Zhang , “Effect of Body Mass Index on Surgical Site Wound Infection, Mortality, and Postoperative Hospital Stay in Subjects Undergoing Possibly Curative Surgery for Colorectal Cancer: A Meta‐Analysis,” International Wound Journal 20, no. 1 (2023): 164–172, 10.1111/iwj.13860.35670494 PMC9797934

The above article, published online on 07 June 2022, in Wiley Online Library (http://onlinelibrary.wiley.com/), has been retracted by agreement between the journal Editor in Chief, Professor Keith Harding; and John Wiley & Sons Ltd. Following an investigation by the publisher, all parties have concluded that this article was accepted solely on the basis of a compromised peer review process. The editors have therefore decided to retract the article. The authors did not respond to our notice regarding the retraction.

